# Efficacy, safety, and tolerability of combined pirfenidone and N-acetylcysteine therapy: a systematic review and meta-analysis

**DOI:** 10.1186/s12890-020-1121-2

**Published:** 2020-05-07

**Authors:** Hanyu Shi, Dawei Yin, Francesco Bonella, Michael Kreuter, Ute Oltmanns, Xuren Li, Shouchun Peng, Luqing Wei

**Affiliations:** 1 Department of Respiratory and Critical Care Medicine, Special Medical Center of Chinese People’s Armed Police Forces, Tianjin, China; 2 Logistics University of Chinese People’s Armed Police Forces, Tianjin, China; 3 Department of Pneumology, Ruhrlandklinik, Centre for Interstitial and Rare Lung Disease, University Hospital, University Duisburg-Essen, Essen, Germany; 4 Center for Interstitial and Rare Lung Diseases, Thoraxklinik, University of Heidelberg, Heidelberg, Germany; 5 Department of Pneumology, Helios Klinikum Pforzheim, Pforzheim, Germany; 6 Department of Respiratory and Critical Care Medicine, Special Medical Center of the Chinese People’s Armed Police Forces, 220, Cheng-Lin Road, Tianjin, China

**Keywords:** Idiopathic pulmonary fibrosis, Pirfenidone, Acetylcysteine, Meta-analysis, Systematic review

## Abstract

**Background:**

While antifibrotic drugs significantly decrease lung function decline in idiopathic pulmonary fibrosis (IPF), there is still an unmet need to halt disease progression. Antioxidative therapy with N-acetylcysteine (NAC) is considered a potential additional therapy that can be combined with antifibrotics in some patients in clinical practice. However, data on the efficacy, tolerability, and safety of this combination are scarce. We performed a systematic review and meta-analysis to appraise the safety, tolerability, and efficacy of the combination compared to treatment with pirfenidone alone.

**Methods:**

We systematically reviewed all the published studies with combined pirfenidone (PFD) and NAC (PFD + NAC) treatment in IPF patients. The primary outcomes referred to decline in pulmonary function tests (PFTs) and the rates of IPF patients with side effects.

**Results:**

In the meta-analysis, 6 studies with 319 total IPF patients were included. The PFD + NAC group was comparable to the PFD alone group in terms of the predicted forced vital capacity (FVC%) and predicted diffusion capacity for carbon monoxide (DLco%) from treatment start to week 24. Side effects and treatment discontinuation rates were also comparable in both groups.

**Conclusion:**

This systematic review and meta-analysis suggests that combination with NAC does not alter the efficacy, safety, or tolerability of PFD in comparison to PFD alone in IPF patients.

## Background

Idiopathic pulmonary fibrosis (IPF), the most common fibrotic interstitial lung disease (ILD), is a chronic, progressive, and irreversible disease characterized by progressive extracellular matrix accumulation leading to respiratory insufficiency. The management strategies for IPF include relieving symptoms, maintaining patient quality of life and slowing disease progression. Apart from non-pharmacological treatments such as long-term oxygen therapy or rehabilitation, antifibrotics are the gold standard and should be started as soon as possible after the diagnosis of IPF [1].

Pirfenidone (PFD), an oral pyridine with antifibrotic, anti-inflammatory and antioxidant functions, is currently approved for the treatment of IPF in most countries and recommended by the latest guidelines [1, 2]. Evidence from the CAPACITY and ASCEND randomized controlled trials (RCTs) showed a significant reduction in the relative decline in forced vital capacity (FVC) over 72 weeks compared to the placebo group [3, 4]. Furthermore, the pooled analysis and meta-analysis suggested a lower relative risk of death in PFD-treated patients than in placebo-treated patients [5]. N-acetylcysteine (NAC), a tripeptide (g-glutamyl-cysteinyl glycine), can replenish glutathione storage levels, increase the antioxidant capacity and correct the imbalance of oxidants and antioxidants associated with fibroproliferation [6]. On the basis of the negative results of the PANTHER trial [7], NAC did not receive a positive recommendation as a treatment for IPF in the latest international guidelines [1, 7].

A substantial number of IPF patients receive combined PFD and NAC therapy [8–10]; however, data on the efficacy, safety, and tolerability of this combination are scarce. A recent placebo-controlled trial (PANORAMA) found that the rate of skin side effects was higher in the PFD + NAC group than in the PFD alone group [11]. However, other studies, including a study with inhaled NAC, suggest a slower lung function decline and a similar side effect profile in patients undergoing PFD + NAC treatment compared with patients undergoing PFD alone [8, 12–14].

Here, we systematically reviewed all studies with combined PFD and NAC treatment in IPF patients and performed a meta-analysis to compare the efficacy, safety, and tolerability of treatment with combined PFD and NAC vs treatment with PFD alone.

## Method and materials

### Literature search

This systematic review and meta-analysis was performed in accordance with the Preferred Reporting Items for Systematic Reviews and Meta-Analyses (PRISMA) statement and the PRISMA 2009-checklist. In addition, the meta-analysis was registered in PROSPERO (registration number: CRD42019134890).

A structured literature search was performed for studies on the safety and efficacy of combined PFD and NAC treatment in IPF patients. The following databases were searched from the earliest available dates to May 2019: PubMed, EMBASE, the Cochrane Library, Ovid, ProQuest, Web of Science and Chinese databases (including the China National Knowledge Infrastructure (CNKI), Chinese VIP Information (VIP), and the Wan Fang database). In addition, “clinicaltrials.gov” and the bibliographies of previous meta-analyses on PFD or NAC were checked for relevant studies. The search terms included “idiopathic pulmonary fibrosis”, “IPF”, and “pulmonary fibrosis” for the disease and “pirfenidone”, “Esbriet”, and “acetylcysteine” for the intervention. No language or research type restriction was adopted.

### Study selection

The inclusion criteria for the meta-analysis were as follows: (1) IPF patients diagnosed according to the 2011 American Thoracic Society/European Respiratory Society (ATS/ERS) guidelines [15]; (2) interventions referring to combined PFD and NAC treatment, regardless of whether administration was oral or inhaled; and (3) the control group consisted of patients who received PFD alone. All appropriate studies were included in the meta-analysis.

Two reviewers (HYS and DWY) inspected all studies after removing duplicate studies by reviewing titles and abstracts. Relevant studies were assessed by viewing the full-text articles to select studies that met the inclusion criteria mentioned above. Disagreements were resolved by a consensus-based discussion.

To collect data on combined PFD + NAC therapy published in observational or retrospective studies involving patients with combined PFD, NAC, and corticosteroid/proton pump inhibitor treatment, we contacted the corresponding authors and obtained the original data regarding PFD + NAC therapy from some studies and excluded those patients receiving glucocorticoids other than PFD + NAC. Other studies with a questionable combined therapy group, incomplete data or an inappropriate control group were excluded [16, 17]. All patients included in the meta-analysis had not received glucocorticoids since the pirfenidone treatment began.

### Data extraction and quality scoring

Two reviewers (HYS and XRL) extracted data from the included studies, including the following baseline characteristics: (1) first author, published year, study type, numbers of patients in the PFD + NAC group and PFD group; (2) changes in pulmonary function test (PFT) parameters such as changes in the predicted forced vital capacity (ΔFVC%) and changes in the predicted diffusion capacity for carbon monoxide (ΔDLco%); and (3) the number of side effects including skin reactions (photosensitivity and skin rash) and gastrointestinal reactions (anorexia, diarrhoea, and reduced appetite); the number of intolerable side effects leading to treatment discontinuation was also recorded.

The quality of the included observational studies was estimated using the Newcastle-Ottawa Quality Assessment Scale (NOS). Two reviewers (HYS and XRL) independently assessed the quality of the included studies in the following three domains: selection, comparability, and outcome. Each study score ranges from 0 to 9 stars in the NOS scoring system [18]. The randomized controlled studies were assessed with the Cochrane Collaboration risk of bias assessment tool [19].

### Data analysis

The data extracted from the selected trials were used to generate forest plots in Stata SE 13.0 software (Stata Corp, College Station, TX, USA). The risk of patients experiencing side effects and other binary parameters are expressed as odds ratios (ORs) for both the included cohort and case-control studies. The changes in the PFT parameters and other continuous parameters are presented as standardized mean differences (SMDs) for different studies that adopted various PFT inclusion standards. We examined the level of heterogeneity to determine which type of analysis to use. If there was low heterogeneity (I^2^ less than 40%), then we used a fixed effects model. If the I^2^ statistic was greater than 40%, we applied a random effects model to summarize the data. Patients with the combination of PFD and inhaled NAC were only included in one case-control study [11], and the sensitivity analysis excluding the case-control study and the secondary analysis with only oral administration studies were completed in one step. Two-tailed *p* values less than 0.05 were considered significant.

## Results

### Study characteristics and quality scores

**Fig. 1:**
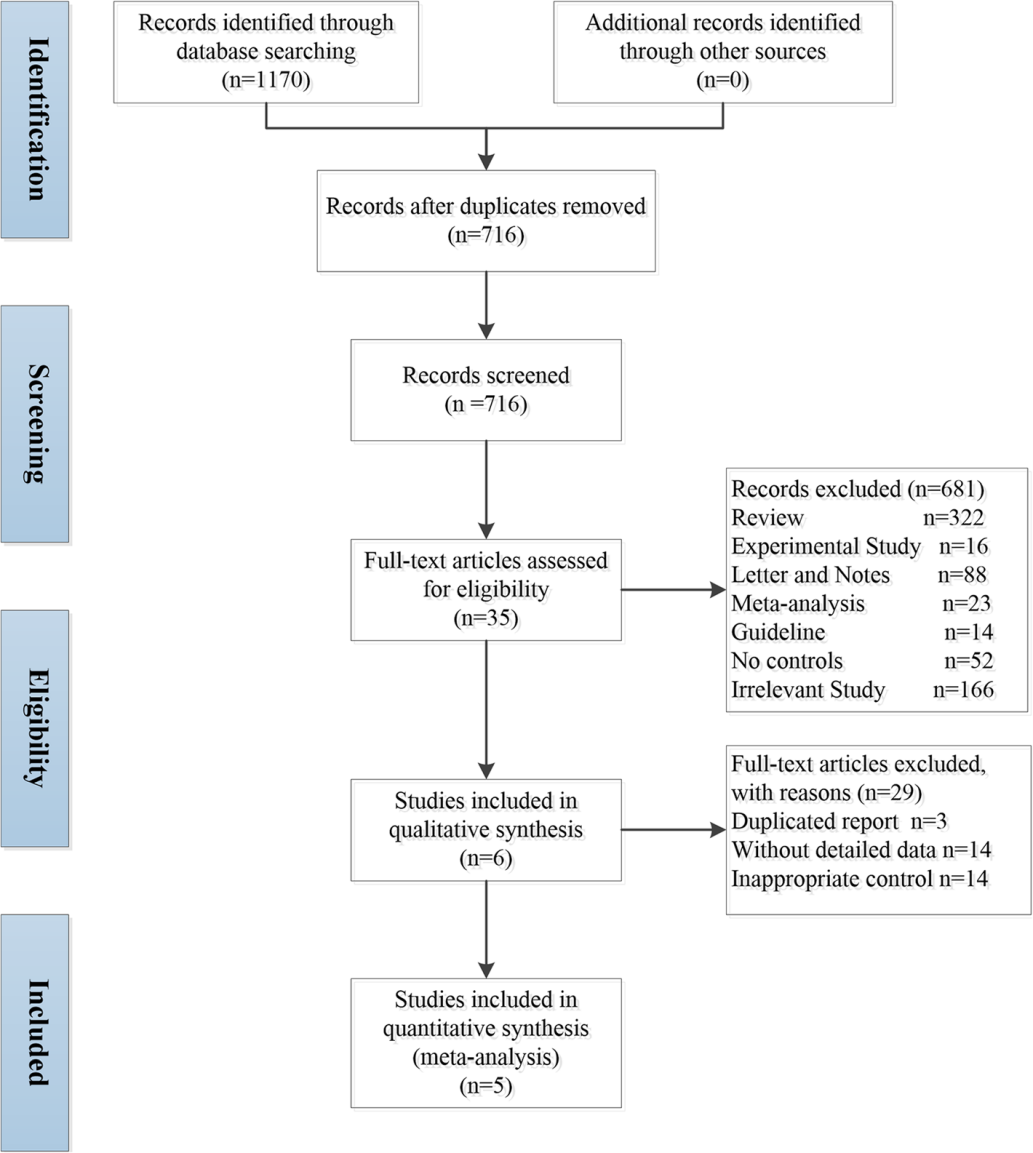
PRISMA flow diagram for the inclusion of studies in the systematic review and meta-analysis

After the removal of duplicates and selection by viewing the abstracts and titles, a full-text review of 35 articles was performed. Six [8, 11–14, 20] and five [8, 12–14, 20] studies were included in the qualitative and quantitative analyses, respectively (Fig. 1). The systematic review comprised a total of 319 patients (PFD + NAC group *n* = 144, PFD alone group *n* = 175). The studies were conducted in Europe (*n* = 4), Japan (*n* = 1) and China (*n* = 2). One study was a controlled clinical trial (PANORAMA trial [11] by Behr et al), four were cohort studies [8, 12, 13, 20], and one was a case-control study [14]. Of note, one RCT was excluded because only the conference abstract was available [21]. A meta-analysis of observational real-world studies including 207 patients was performed. The general characteristics of the studies are shown in Table 1.

**Table 1:** Baseline characteristics of studies in the systematic review and meta-analysis

Study ID	Number of PFD + NAC/PFD Patients	Age (years) (PFD + NAC/PFD)	Country/Study Area	Study Type	Daily Dosage of PFD + NAC [mg/d]	Daily Dosage of PFD [mg/d]	Outcome Parameters
Bonella 2013 [8]	6/16	71.3 ± 7.50/70.5 ± 6.4	Germany	Cohort	1200–1800 + 1800	1200–1800	1, 2, 3, 4, 5, 6
Oltmanns 2014 [20]	11/45	68 ± 6.6/68 ± 7.9	Germany	Cohort	2400 + 1800	2400	1, 4, 5, 6
Mao 2018 [13]	35/33	59.8 ± 13.4/58.2 ± 9.0	China	Cohort	1800 + 600	1800	1, 2, 3
Ma 2018 [12]	15/15	62.1 ± 6.6/64.9 ± 4.3	China	Cohort	1800 + 1200	1800	1, 2, 3, 5, 6
Sakamoto 2014 [Inhaled] [14]	17/10	73.5/75.0^a^	Japan	CC	1800 + 704.8	1800	1, 2, 3, 4, 5
Behr 2016 [11]	60/62	66.7/67.5^a^	Europe	RCT	1602–2403 + 1800	1602–2403	1, 2, 3, 4, 5, 6

*Abbreviations*: *PFD* Pirfenidone, *NAC* N-acetylcysteine, *CC* Case-control study, *Cohort* Cohort study, *RCT* Randomized controlled trial. ^a^Median for ages. Outcome parameters legend: 1: At least one side effect; 2: Gastrointestinal side effects; 3: Skin side effects; 4: Intolerable side effects; 5: Decline in forced vital capacity percent predicted (ΔFVC%) from treatment start; and 6: Decline in diffusion capacity for carbon monoxide (ΔDLco%) from treatment start

The average quality score for the included observational studies was 7.25 for cohort studies and 6 for the case-control study based on the NOS. The only RCT, which was conducted by Behr et al. [11], had high quality after being assessed according to the Cochrane Collaboration risk of bias assessment tool. The detailed quality characteristics are shown in the Table S1.

### Effect of combined pirfenidone and acetylcysteine therapy on lung function parameters

**Fig. 2:**
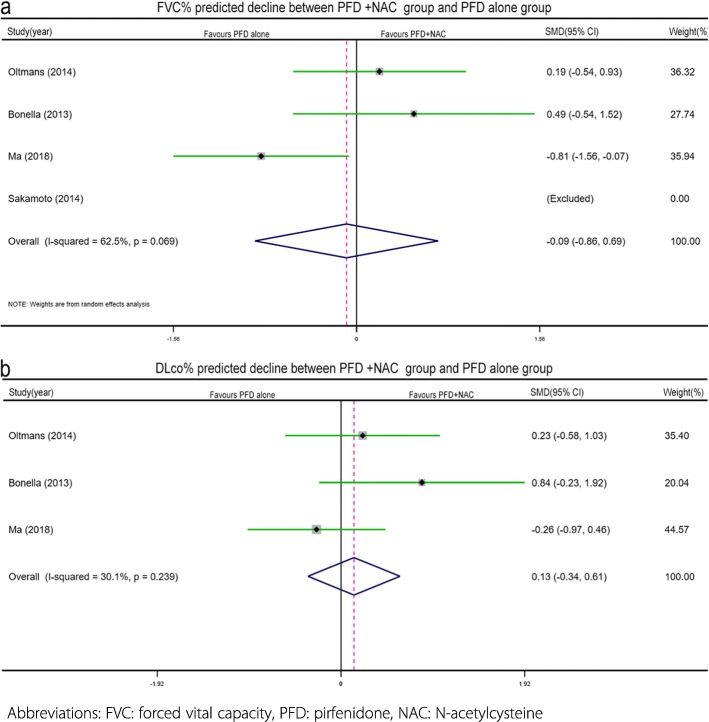
Forest plot of efficacy profile (outcomes: the predicted decline in FVC% (**a**) and DLco% (**b**) between the combined pirfenidone and acetylcysteine group and the pirfenidone alone group

The ΔFVC% predicted from baseline to week 24 was available in four studies with a total of 108 patients (PFD + NAC: *n* = 48, PFD alone: *n* = 60). Due to the lack of standard deviation values provided, the study by Sakamoto et al. [14] was excluded. Therefore, only three studies [8, 12, 20] were included in the meta-analysis. Given the premise of moderate heterogeneity (I^2^ = 62.5%, *p* = 0.069), the random effects model was applied for the analysis. The results showed that PFD + NAC therapy had no additional benefit in reducing the decrease in lung function (SMD = -0.09, 95% CI − 0.86-0.69, *p* = 0.295, Fig. 2a) compared to PFD alone.

Similarly, the ΔDLco% predicted from baseline to week 24 was available in 3 studies [8, 12, 20] with a total of 76 patients (PFD + NAC: *n* = 28, PFD alone: *n* = 48). These studies had low heterogeneity (I^2^ = 30.1%, *p* = 0.239). There was no difference in the ΔDLco% between the PFD + NAC group and the PFD group (SMD = 0.13, 95% CI -0.34-0.61, *p* = 0.580, Fig. 2b).

### Safety and treatment tolerability of combined pirfenidone and acetylcysteine therapy

**Fig. 3:**
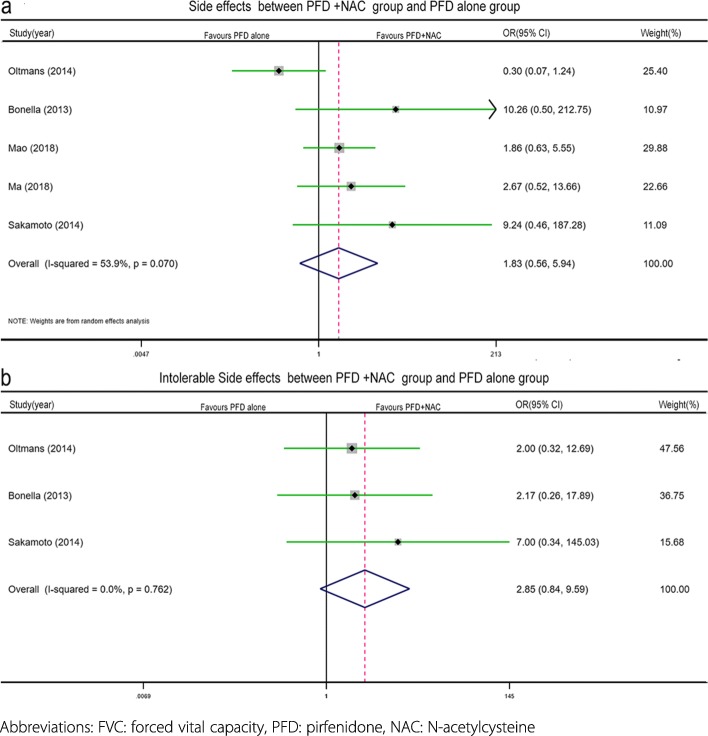
Forest plot of the safety profile (outcome measure: at least one side effect, (**a**)) and tolerability profile (outcome measure: intolerable side effects leading to treatment discontinuation, (**b**)) between the combined pirfenidone and acetylcysteine group and the pirfenidone alone group

**Fig. 4:**
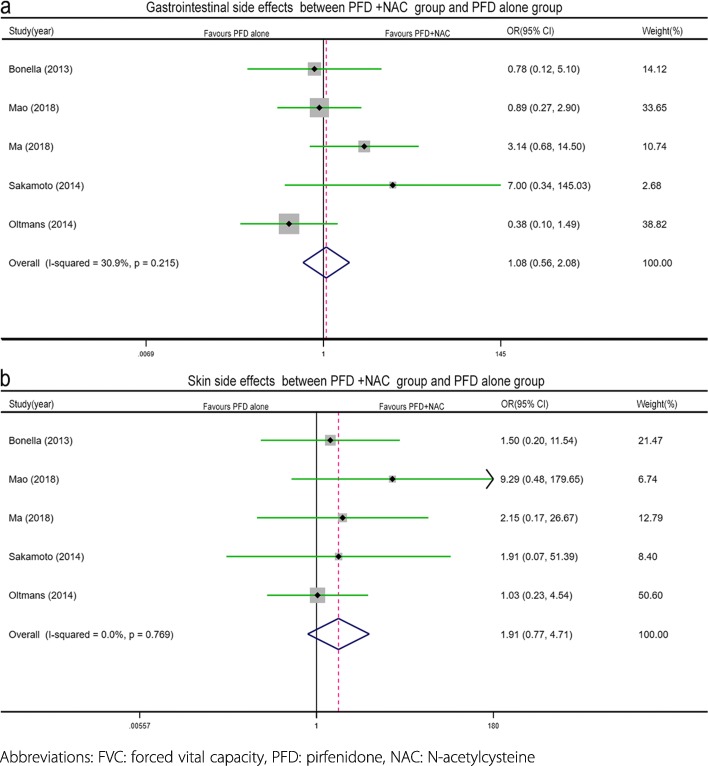
Forest plot of the specific safety profile (outcome measure: gastrointestinal side effects (**a**) and skin side effects (**b**)) between the combined pirfenidone and acetylcysteine group and the pirfenidone alone group

The number of patients who experienced at least one side effect was mentioned in five studies, including a total of 207 patients [8, 12–14, 20] (PFD + NAC: *n* = 84, PFD alone: *n* = 113). Moderate significant heterogeneity (I^2^ = 53.9%, *p* = 0.070) was detected, and a random effects model was applied. The results suggested that the rate of at least one side effect in the PFD + NAC therapy group was similar (PFD + NAC vs PFD alone: 41 vs 57, OR = 1.83, 95% CI 0.56–5.94, *p* = 0.314, Fig. 3a) to that in the PFD alone group. No significant differences were observed in the rates of specific side effects (PFD + NAC vs PFD alone: gastrointestinal (GI): 26 vs 47, I^2^ = 30.9%, *p* = 0.215, OR = 1.08, 95% CI 0.56–2.08, *p* = 0.811, Fig. 4a; skin side effects: 12 vs 17, I^2^ = 0%, *p* = 0.769, OR = 1.91, 95% CI 0.77–4.71, *p* = 0.162, Fig. 4b) between the treatment groups in the subgroup analysis.

Intolerable side effects leading to treatment discontinuation were reported in three studies with a total of 100 patients [8, 14, 20] (PFD + NAC *n* = 34, PFD alone *n* = 66). There was no significant heterogeneity (I^2^ = 0%, *p* = 0.762) observed among these studies. The results showed that combined PFD + NAC therapy did not increase the risk of intolerable side effects (OR = 2.85, 95% CI 0.84–9.59, *p* = 0.092, Fig. 3b) in comparison with PFD therapy. Patients receiving PFD + NAC therapy experienced intolerable side effects at a similar frequency as in those receiving PFD alone.

### Qualitative analysis and sensitivity analysis

Funnel plots and Egger’s test could not be used to check for the existence of publication bias because our meta-analysis included fewer than 10 studies [19]. In addition, the secondary meta-analysis with only oral NAC studies and sensitivity analysis excluding the case-control study resulted in *p* values of 0.249, 0.611 and 0.955 for gastrointestinal, skin and intolerable side effects, respectively, and the forest plots composed of only oral NAC studies can be found in the Supplement Material (Figures S1, S2 and S3). In the ensuing quantitative analysis comparing the results of the meta-analysis and Behrs’ RCT (Table 2), the safety, tolerability, and efficacy outcomes [11] were similar in the PFD + NAC treatment group and the PFD monotherapy group except for a significantly higher rate of skin adverse effects in the RCT (p values in meta vs RCT: 0.097/0.038). Other parameters, such as the six-minute walk distance (6MWD) and progression-free survival (PFS), were available in only one study. The 6MWD results were comparable between the observational study (− 13.25 ± 6.77 vs − 16.59 ± 4.65, *p* = 0.159) [12] and Behr’s RCT (− 4.3 vs − 11.7, *p* = 0.54). In addition, PFD + NAC treatment showed favourable results regarding the PFS (median survival days 304 d vs 168 d; *p* = 0.016) in the case-control study [14].

**Table 2:** The 95% confidence intervals and *P* values of different parameters in the meta-analysis, meta-analysis without the case-control study, and Behr’s RCT

	Meta-analysis (Oral and Inhaled studies)		Meta-analysis (Oral studies only)		Behr’s RCT	
	95% CI (OR/SMD)	*P* values	95% CI (OR/SMD)	*P* values	95% CI (OR/SMD)	*P* values
SE	0.56–5.94	0.314	0.43–5.17	0.068	0.33–1.88	0.592
GI_SE	0.56–2.08	0.811	0.46–1.83	0.249	0.33–1.68	0.475
Skin_SE	0.77–4.71	0.162	0.74–4.88	0.611	1.14–77.52	0.038
Intole_SE	0.84–9.59	0.092	0.52–8.32	0.955	0.31–6.78	0.635
ΔFVC%	-0.86-0.69	0.693	NA	NA	−4.23-0.13	0.200
ΔDLco%	−0.34-0.62	0.580	NA	NA	−2.44-2.22	0.730

*Abbreviations*: *SE* Side effect, *GI_SE* Gastrointestinal side effects, *Intole_SE* Intolerable side effects (leading to treatment discontinuation); ΔFVC%: decline in forced vital capacity percent predicted from treatment start to week 24; ΔDLco%: decline in diffusion capacity for carbon monoxide from treatment start to week 24

## Discussion

The present meta-analysis did not show superior efficacy of the combination PFD plus NAC therapy in slowing lung functional decline in IPF and showed comparable safety and tolerability compared to PFD alone.

The antifibrotic drug pirfenidone can significantly reduce lung functional decline in IPF patients; therefore, it is recommended in international guidelines as the treatment of choice [1]. However, patients still present with gradually worsening symptoms and a constant loss of quality of life [22], and the outcome is comparable to those of many malignant diseases [23]. There is still an unmet need to halt disease progression. Antioxidative therapy with NAC is discussed as a potential additional therapy in some patients in clinical practice.

The randomized placebo-controlled trial IFIGENIA investigated NAC treatment vs the standard treatment with prednisone plus azathioprine in 182 mild to moderate IPF patients over 48 weeks [24]. Combined therapy with high-dose NAC (1800 mg, d), prednisone and azathioprine significantly preserved the absolute vital capacity (VC) and DLco compared to the combination of prednisone and azathioprine [24]. However, the results of the PANTHER-IPF trial [25], which also enrolled patients with mild to moderate IPF, showed that there was no significant difference in the decline in FVC and showed a higher rate of serious adverse effects [25] and especially a higher mortality rate in patients receiving triple therapy than in patients receiving the placebo. While another report of the PANTHER also demonstrated no benefit of NAC over the placebo [7], a post hoc analysis of the PANTHER study [26] suggested that the genotypic background of IPF patients may have an impact on the effects of NAC treatment. MUC5B and TOLLIP SNPs were retrospectively investigated in a subgroup of patients in the PANTHER trial. Patients with a rs3750920 (TOLLIP) TT genotype (25% of all patients) showed favourable outcomes regarding a reduction in the risk of the composite endpoint, defined as death, transplant, hospitalization or ≥ 10% FVC decline, while patients with a CC genotype had a non-significant increase in the composite physiological index (CPI) [26].

Regarding lung function decline (especially FVC), our meta-analysis demonstrated comparable outcomes between the PFD + NAC group and PFD monotherapy group. Considering that the majority of studies included in this meta-analysis enrolled Caucasian patients with mild to moderate IPF (predicted FVC from 50 to 90%), the heterogeneity among these studies may be related to ethnicity because the studies by Ma and Sakamoto [12, 14], which showed favourable efficacy results for the combination treatment, enrolled Asian patients. In addition, a speculative explanation for this phenomenon could be that the proportion of patients with the TOLLIP TT genotype in the treatment groups differed among the studies, but the data were not available [7, 26]. Furthermore, direct antioxidant and anti-inflammatory effects on the alveoli by inhaled instead of oral NAC treatment may also contribute to the favourable outcomes in Sakamoto’s study [14].

There are some considerations regarding the safety and tolerability of PFD and NAC treatment in IPF patients. Gastrointestinal (diarrhoea, anorexia, etc.) and skin side effects (photosensitivity and skin rash) are the most common adverse effects experienced by IPF patients receiving PFD treatment [27, 28]. Compared to the findings from the PANORAMA trial, our meta-analysis showed a similar rate of side effects except for skin side effects (lower rate). The exact reason for this difference is unclear but may be related to differences in the patients’ habits, such as the time spent outdoors or the use of skin protection creams [11].

Our meta-analysis has several limitations. First is the small number of included studies. Second, the meta-analysis included only one RCT, and the rest of the studies were observational studies and real-world experiences. Third, the lung function decline assessment was partial because scarce data were available for the 6MWD and blood gas analysis; therefore, we cannot exclude improvements in other outcome measures due to treatment with combined PFD + NAC. Fourth, the random effects model, which is generally used to analyse the overall effect when moderate heterogeneity exists (I^2^ > 40%), was applied for the analysis of patients experiencing at least one side effect and to assess differences in the FVC% decline between groups, leading to a wider confidence interval and a more conservative conclusion.

## Conclusions

In conclusion, this systematic review and meta-analysis suggests that the combination of PFD and NAC does not alter the efficacy, safety, or tolerability of PFD in comparison to PFD alone in the IPF study population. High-quality, multi-centre RCTs and large-sample real-world observational studies evaluating the safety, tolerability, and efficacy of PFD + NAC therapy vs PFD monotherapy and investigating the genetic background of patients are needed to validate these results.

## Supplementary information

**Additional file 1: Table S1.** Quality scores of observational studies in the meta-analysis based on NOS scoring system. **Figure S1.** Forest plot of efficacy profile (outcomes: the predicted decline in FVC% (Figure S1-a) and DLco% (Figure S1-b)) between the combined pirfenidone and acetylcysteine group and the pirfenidone alone group with only oral NAC studies. Abbreviations: FVC: forced vital capacity, PFD: pirfenidone, NAC: N-acetylcysteine. **Figure S2.** Forest plot of the safety profile (outcome measure: at least one side effect, Figure S2-a) and tolerability profile (outcome measure: intolerable side effects leading to treatment discontinuation, Figure S2-b) between the combined pirfenidone and acetylcysteine group and the pirfenidone alone group with only oral NAC studies. Abbreviations: PFD: pirfenidone, NAC: N-acetylcysteine. **Figure S3.** Forest plot of the specific safety profile (outcome measure: gastrointestinal side effects (Figure S3-a) and skin side effects (Figure S3-b)) between the combined pirfenidone and acetylcysteine group and the pirfenidone alone group with only oral NAC studies. Abbreviations: PFD: pirfenidone, NAC: N-acetylcysteine.

## Data Availability

The datasets used and/or analyzed during the current study are available from the corresponding author on reasonable request.

## Abbreviations

IPF: Idiopathic pulmonary fibrosis
ILD: Interstitial lung disease
NAC: N-acetylcysteine
PFD: Pirfenidone
PFT: Pulmonary function test
FVC: Forced vital capacity
ΔFVC%: Changes in predicted forced vital capacity
DLco: Diffusion capacity for carbon monoxide
ΔDLco%: Changes in the predicted diffusion capacity for carbon monoxide
RCT: Randomized controlled trials
SNP: Single nucleotide polymorphism
PRISMA: Preferred Reporting Items for Systematic Reviews and Meta-Analyses
PROSPERO: International prospective register of systematic reviews
NOS: Newcastle-Ottawa Quality Assessment Scale
ATS/ERS: American Thoracic Society/European Respiratory Society
ORs: Odds ratios
SMDs: Standardized mean differences
6MWD: Six min walk distance
PFS: Progression-free survival
