# Supplementary material for: Efficacy, safety, and tolerability of combined pirfenidone and N-acetylcysteine therapy: a systematic review and meta-analysis
Source: BMC Pulm Med. 2020 May 7;20:128. doi: 10.1186/s12890-020-1121-2 (PMC7204217; doi:10.1186/s12890-020-1121-2)
Supplement: Supplementary file 1 — Additional file 1: Table S1. Quality scores of observational studies in the meta-analysis based on NOS scoring system. Figure S1. Forest plot of efficacy profile (outcomes: the predicted decline in FVC% (Figure S1-a) and DLco% (Figure S1-b)) between the combined pirfenidone and acetylcysteine group and the pirfenidone alone group with only oral NAC studies. Abbreviations: FVC: forced vital capacity, PFD: pirfenidone, NAC: N-acetylcysteine. Figure S2. Forest plot of the safety profile (outcome measure: at least one side effect, Figure S2-a) and tolerability profile (outcome measure: intolerable side effects leading to treatment discontinuation, Figure S2-b) between the combined pirfenidone and acetylcysteine group and the pirfenidone alone group with only oral NAC studies. Abbreviations: PFD: pirfenidone, NAC: N-acetylcysteine. Figure S3. Forest plot of the specific safety profile (outcome measure: gastrointestinal side effects (Figure S3-a) and skin side effects (Figure S3-b)) between the combined pirfenidone and acetylcysteine group and the pirfenidone alone group with only oral NAC studies. Abbreviations: PFD: pirfenidone, NAC: N-acetylcysteine. [file 12890_2020_1121_MOESM1_ESM.docx]

**Table S1:** Quality scores of observational studies in the meta-analysis based on NOS scoring system

| Study ID | Study Type | Selection | Comparability | Outcome/Exposure | Total Scores |
| --- | --- | --- | --- | --- | --- |
| Oltmanns2014 | Cohort | ★★★★ | ★ | ★★ | 7 |
| Bonella2013 | Cohort | ★★★★ | ★ | ★★ | 7 |
| Mao2018 | Cohort | ★★★★ | ★ | ★★★ | 7 |
| Ma2018 | Cohort | ★★★★ | ★★ | ★★ | 8 |
| Sakamoto2014 | CC | ★★★ | ★ | ★★ | 6 |

Abbreviations: NOS: Newcastle-Ottawa quality assessment scale, Cohort: Cohort study, CC: Case-control study


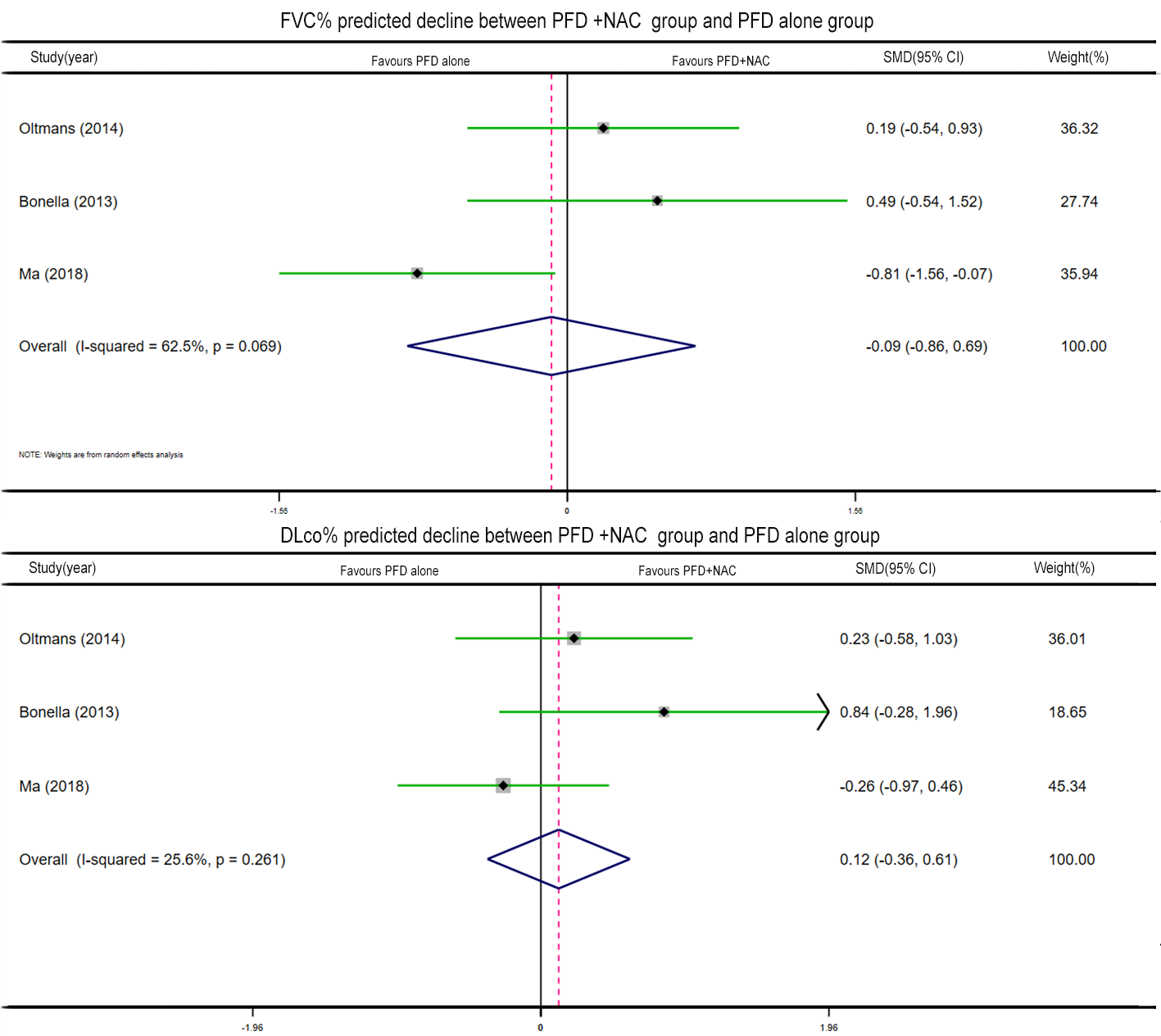


**Figure S1** Forest plot of efficacy profile (outcomes: the predicted decline in FVC% (Figure S1-a) and DLco% (Figure S1-b)) between the combined pirfenidone and acetylcysteine group and the pirfenidone alone group with only oral NAC studies. FVC: forced vital capacity, PFD: pirfenidone, NAC: N-acetylcysteine.


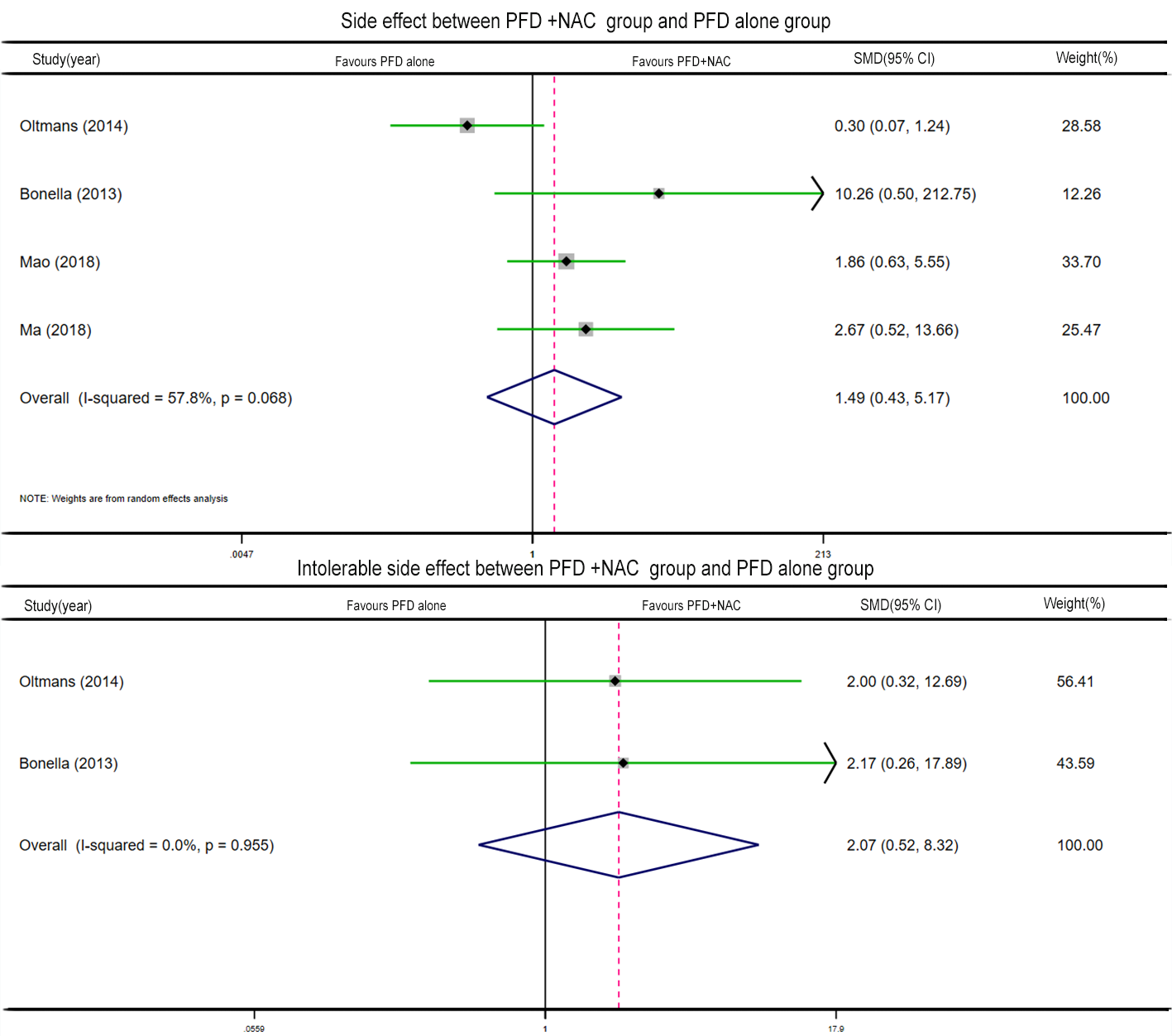


**Figure S2** Forest plot of the safety profile (outcome measure: at least one side effect, Figure S2-a) and tolerability profile (outcome measure: intolerable side effects leading to treatment discontinuation, Figure S2-b) between the combined pirfenidone and acetylcysteine group and the pirfenidone alone group with only oral NAC studies. PFD: pirfenidone, NAC: N-acetylcysteine.


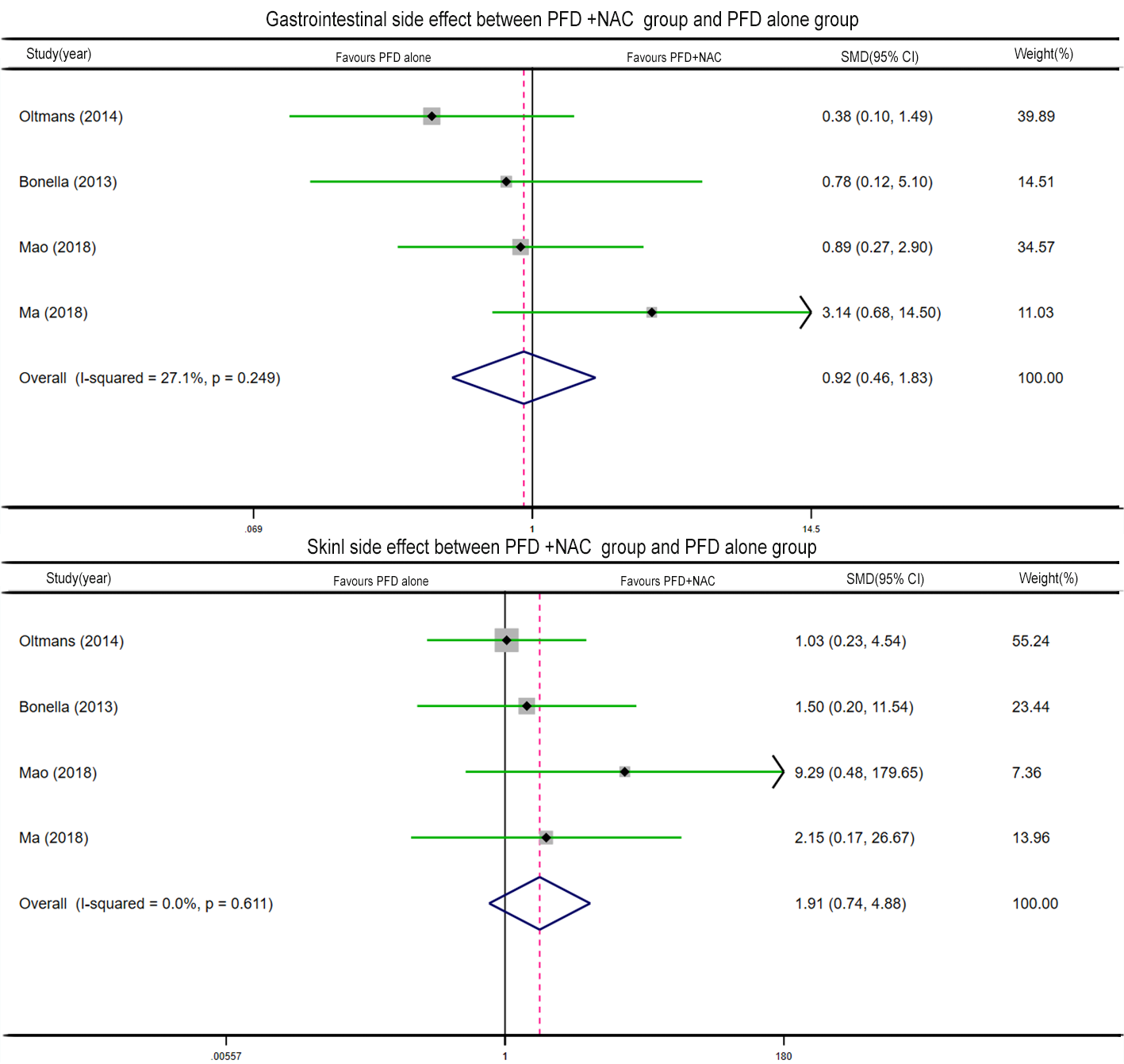


**Figure S3** Forest plot of the specific safety profile (outcome measure: gastrointestinal side effects (Figure S3-a) and skin side effects (Figure S3-b)) between the combined pirfenidone and acetylcysteine group and the pirfenidone alone group with only oral NAC studies. PFD: pirfenidone, NAC: N-acetylcysteine.
